# A high-performance liquid chromatography-electronic circular dichroism online method for assessing the absolute enantiomeric excess and conversion ratio of asymmetric reactions

**DOI:** 10.1038/srep43278

**Published:** 2017-03-02

**Authors:** Xiang Zhang, Mingchao Wang, Li Li, Dali Yin

**Affiliations:** 1 State Key Laboratory of Bioactive Substances and Functions of Natural Medicines, Beijing Key Laboratory of Active Substances, Discovery and Druggability Evaluation, Institute of Materia Medica, Peking Union Medical College & Chinese Academy of Medical Sciences, Beijing, China.

## Abstract

Asymmetric reactions often need to be evaluated during the synthesis of chiral compounds. However, traditional evaluation methods require the isolation of the individual enantiomer, which is tedious and time-consuming. Thus, it is desirable to develop simple, practical online detection methods. We developed a method based on high-performance liquid chromatography-electronic circular dichroism (HPLC-ECD) that simultaneously analyzes the material conversion ratio and absolute optical purity of each enantiomer. In particular, only a reverse-phase C18 column instead of a chiral column is required in our method because the ECD measurement provides a *g*-factor that describes the ratio of each enantiomer in the mixtures. We used our method to analyze the asymmetric hydrosilylation of β-enamino esters, and we discussed the advantage, feasibility, and effectiveness of this new methodology.

Chiral natural products are usually present as single enantiomers, rather than racemic mixtures, and many chiral drugs also have enantiomers. Different stereoisomers often demonstrate distinct pharmacological, toxicological, and metabolic properties. Consequently, the US Food and Drug Administration require that the pure enantiomers of chiral drugs must be evaluated^1^. The preference for optically pure products means that the development of asymmetric reactions has been a frontier field in organic chemistry for several decades, and appeals many talented organic scientists to discover novel reactions and reagents^2^,^3^,^4^.

To evaluate an asymmetric reaction, the conversion ratio and the product’s optical purities must be measured precisely. Currently, the desired product containing both enantiomers is isolated from the reaction mixture to obtain the product yield. Next, the purified product is separated by high-performance liquid chromatography (HPLC) with a suitable chiral column to obtain the pure optical isomers. The enantiomeric excess (*ee*) of a product can be calculated according to the peak area or height ratios^5^. However, the absolute configuration of the main isomer is still unknown, although this information is crucial. Two classical methods are often used to measure the absolute configuration of the optical isomers, which are obtained by chiral separation. The first is single-crystal X-ray diffraction of the purified isomers, which requires a high-quality crystal. The second is chemical correlation with compounds that have known absolute configurations. These methods are tedious, time-consuming, and difficult, and they cannot be used for online stereochemical assignment.

Electronic circular dichroism (ECD), which records the differential absorption between left- and right-circularly polarized light of a compound, is widely used in stereochemical studies of chiral molecules in combination with quantum chemical calculations^6^,^7^,^8^,^9^,^10^,^11^. HPLC-ECD analysis is expected to provide a stereochemical assignment for each component of racemic compounds, and offer important information about the absolute configuration and *ee* value of an unknown sample, even at a single wavelength^12^,^13^. Several groups have measured the chemical and enantiomeric purities of synthesis products with an ECD online detector coupled with HPLC using a non-chiral stationary phase column^14^,^15^. However, to the best of our knowledge, there is no report of reaction optimization by HPLC-ECD.

In this work, we develop a direct, rapid, online analytical method to detect the material conversion ratio instantly, and simultaneously calculate the absolute optical purity of the target isomer by combining HPLC analysis and ECD detection.

## Results and Discussion

### Model selection

**Figure 1:**
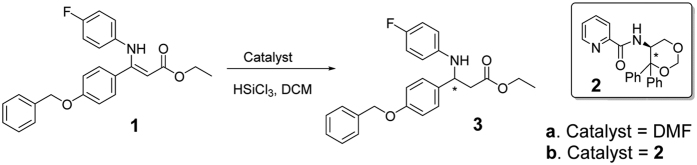
Hydrosilylation of a β-enamino ester. The reaction was conducted in two different procedures: (**a**) N,N-dimethylformamide (DMF) was used as an achiral catalyst and the product was racemic; (**b**) compound **2** was used as the chiral catalyst and the product was enantioselective.

A reaction that is involved in the new synthetic strategy for ezetimibe^16^, a new type of cholesterol absorption inhibitor, was chosen to verify the feasibility and effectiveness of our method. The process for large-scale preparation of this compound is being developed. In this synthetic procedure, the asymmetric hydrosilylation of a β-enamino ester (Fig. 1) catalysed by a Lewis base^17^,^18^,^19^,^20^,^21^,^22^ is the key step in constructing the chiral centre of ezetimibe. It is important to monitor the reaction in real time, so online detection is necessary. Thus, we selected this reaction to test our method.

### Development and validation of the method

**Figure 2:**
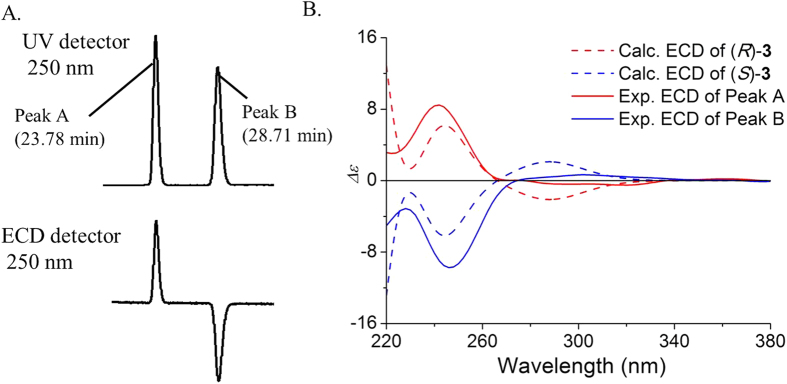
(**A**) Resolution of *rac*-**3** on the chiral OD-H column monitored at 250 nm (top: UV, bottom: ECD). These two compounds show nearly mirror-image ECD curves with intense CEs. (**B**) Comparison of calculated ECD spectra of enantiomeric **3** with online ECD spectra. The calculated ECD curves show that *R* configuration has a strong positive CE at 250 nm, thus peak A corresponds to the *R* configuration. Similarly, peak B corresponds to the *S* configuration.

The preliminary study of this reaction was performed with DMF as an achiral Lewis base catalyst (Fig. 1, Procedure **a**). A pair of racemic products ((*R*)- and (*S*)-**3**) was generated and applied to an OD-H chiral column, which gave two equal peaks at 23.78 (Peak A) and 28.71 min (Peak B) (Supplementary Figure S1). These two components showed nearly mirror-image ECD curves with intense Cotton effects (CEs) at 244 nm and weak CEs at 300 nm (Fig. 2). At this point, the absolute configuration of each component was still unclear.

The absolute configurations were assigned by comparing the experimental and calculated ECD spectra obtained by using quantum-chemical calculations and time-dependent density functional theory (TDDFT). The theoretical results showed that the *R* configuration of **3** possessed a strong positive CE at 250 nm and a weak negative CE at around 300 nm, and *vice versa* for the *S* configuration. Thus, peak A at 23.78 min corresponds to the *R* configuration and peak B at 28.71 min corresponds to the *S* configuration.

**Figure 3:**
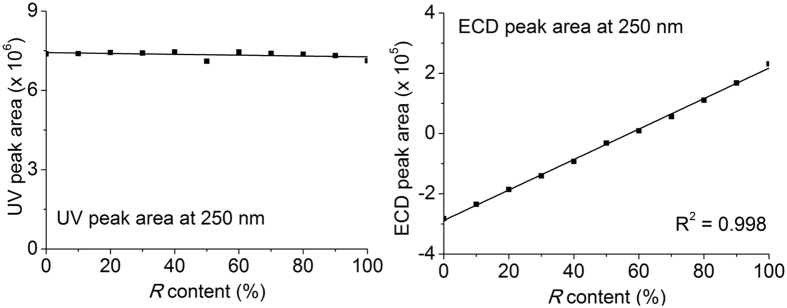
Correlation of the UV and ECD signals with the *R* content using an OD-H chiral column. The UV absorption remained constant and the sum of the ECD peak areas of the two enantiomers varied linearly with the *R* content in the mixture.

To determine the linear concentration range, we constructed standard curves for the pure enantiomers of **3** (Supplementary Figure S2). The pure enantiomers of **3** were obtained by separating racemic products through a preparative chiral OD-H column. The pure enantiomers were mixed in different ratios at a fixed concentration, which was selected from the linear concentration range. These samples were analysed using a chiral OD-H column. The UV absorption remained constant and the sum of the ECD peak areas of the two enantiomers varied linearly with the *R* content in the mixture (Fig. 3). This result indicates that the optical purity can be determined from the ECD peak area ratio of enantiomers instead of the commonly used UV area ratio.

Organic reactions are often monitored by HPLC analysis with a reverse-phase column. Thus, analysis combining reverse-phase HPLC and an ECD detector (RPLC-ECD) could provide information on both the reaction conversion and optical purity of the target compound. Based on this idea, mixtures of (*R*)- and (*S*)-**3** were analysed by HPLC with a commonly used C18 reverse-phase column. As expected, the standard absorption intensity curves and ECD peak areas were consistent with the results obtained from the OD-H column.

**Figure 4:**
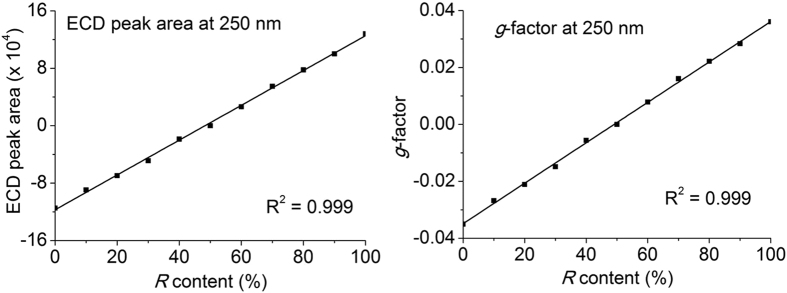
Correlation of *g*-factor and ECD signal with the *R* content using a C18 reverse-phase column. Both ECD signal and *g*-factor changed in line with the *R* content in the mixture.

Because it is difficult to determine the concentration of samples accurately from a reaction mixture directly, the anisotropy factor (*g*-factor) was used to remove the effect of concentration. The *g*-factor is also known as Kuhn’s dissymmetry ratio, which is the ratio of the ECD signal to the light absorbance signal^13^. For the pure enantiomer, the *g*-factor remained constant at 250 nm over the linear concentration range (Supplementary Figure S3). However, both the ECD signals and the *g*-factor changed with the *R* content (Fig. 4). These results indicate that the conversion ratio and absolute optical purity could be obtained by a single injection of samples taken from a reaction mixture following a simple workup. This will be a rapid, effective method to provide information on the absolute configuration and content of enantiomers.

### Method application

Based on our results, we applied this method to the optimization of the asymmetric hydrosilylation of a β-enamino ester (Fig. 1, Procedure **b**) to confirm the robustness of the technique. (*S*)-*N*-(4,4-Diphenyl-1,3-dioxan-5-yl)picolinamide (**2**) was selected as the catalyst for this reaction^22^.

An orthogonal array design is often used in optimization to minimize the number of experimental cycles^23^. We used this design to determine the roles of three variable parameters in the reduction, including the amount of chiral Lewis base catalyst (A), temperature (B), and reaction time (C). For each factor, three levels were given in an L_9_(3^4^) orthogonal array (Table 1)^24^,^25^,^26^.

The experimental results and analysis of the L_9_(3^4^) orthogonal array experiment are presented in Tables 2 and 3. Conventionally, the products would be applied to a C18 column to obtain the conversion ratio, or to a suitable chiral column to obtain the relative optical purity.

Convincing results were obtained with our method, even after adding the reaction mixture to water and analyzing the organic layer directly (Supplementary Figure S4). In Table 3, parameter M is the statistical average of the conversion ratio or optical purity at one level (for one factor). Parameter R is the statistical range of M1–M3 for one factor. Different values of M show the effects of each level on the conversion ratio or optical purity. A higher M value indicates a higher level for the factor. According to the range values for conversion ratio, the three influencing factors were in the order A (catalyst) > C (time) > B (temperature). The optimal combination was A_2_B_3_C_3_, which did not appear in the nine experiments, is closest to A_2_B_2_C_3_ in the orthogonal experiment. Based on the same rule, the three influencing factors for improving the optical purity of product were in the order A (catalyst) > B (temperature) > C (time). The optimal reaction conditions were −10 °C for 8 h with 5 mol % chiral Lewis base catalyst. These data and information verified the efficiency of the method.

Chiral separation and method validation are not required to apply this method to reaction optimization. They were performed in this work to demonstrate the principle and reliability of the method. For a new reaction, stereochemical information about the product could be obtained from the sign and relative intensity of the *g*-factors obtained from a single injection of the reaction mixture. For optimization, the main concern is the relative enantiomeric selectivity, and the exact value is not important. Thus, our method could be applied to a high-throughput reaction optimization study without prior validation.

## Conclusions

We have developed a practical online enantioselective detection method for monitoring asymmetric catalytic reactions. The method provided the material conversion ratio and absolute optical purity of the product simultaneously by using RPLC-ECD without tedious separation or purification procedures. Furthermore, a conventional reverse-phase C18 column was used instead of a chiral column. Our method will liberate chemists from having to find suitable chiral columns by trial and error.

## Experimental Section

### General information

NMR spectra were recorded on NMR spectrometers (Mercury-400 or Mercury-500, Varian) in CDCl_3_ or DMSO-*d*_*6*_ using TMS as the internal standard. The following multiplicity abbreviations are used: singlet (s), doublet (d), triplet (t), quartet (q), and multiplet (m). FT-IR spectra were measured on an FT-IR microscope (Nicolet 5700, Thermo Fisher Scientific). ESI-HRMS data were collected on a mass spectrometer (Exactive Orbitrap, Thermo Fisher Scientific). HPLC-ECD analysis was carried out on an HPLC system (LC-2000, Jasco), consisting of a diode-array detector (MD-2010, Jasco), quaternary gradient pump (PU-2089, Jasco), and autosampler (AS-2055, Jasco), connected to an ECD detector (CD-2095, Jasco). The detection wavelength was set at 250 nm. Separation was performed on an OD-H chiral column (Daicel, 5 μm, 4.6 × 250 mm) or a C18 column (Ultimate XB-C18, Welch, 5 μm, 4.6 × 250 mm). Melting points were determined on a microscope melting point apparatus (MP-J3, Yanaco). Anhydrous dichloromethane (DCM) was prepared by a solvent purification system (PS-MD-7, Inert). Flash column chromatography was performed on flash chromatography system (Isolera One, Biotage). For NMR, IR and HRMS spectra of the synthesized compounds in this article, see Supplementary Figures S5–17.

Ethyl (*Z*)-3-(4-(benzyloxy)phenyl)-3-((4-fluorophenyl)amino) acrylate (**1**) and (*S*)-*N*-(4,4-diphenyl-1,3-dioxan-5-yl)picolinamide (**2**) were synthesized according to a literature method^22^. The spectroscopic data confirmed their structures.

Compound **1**: mp: 72–74 °C. ^1^H NMR (400 MHz, CDCl_3_): *δ* 10.20 (s, 1 H), 7.38 (m, 6 H), 7.25 (m, 1 H), 6.87 (m, 2 H), 6.80 (m, 2 H), 6.66 (m, 2 H), 5.04 (s, 2 H), 4.97(s, 1 H), 4.20 (q, *J* = 7.2 Hz, 2 H), 1.31 (t, *J* = 7.2 Hz, 3 H). ^13^C NMR (100 MHz, CDCl_3_): *δ* 170.3, 160.1, 159.8, 159.1, 157.7, 136.8, 136.8, 136.5, 129.8, 128.7, 128.2, 128.1, 127.6, 124.1, 124.0, 115.5, 115.3, 114.8, 114.8, 90.3, 70.1, 59.3, 14.6. FT-IR *ν*_max_/cm^−1^: 3246, 3088, 3037, 2982, 2926, 2903, 2873, 1651, 1617, 1594, 1513, 1034, 1021. ESI-HRMS (*m*/*z*): [M + H]^+^ calcd for C_24_H_23_O_3_NF 392.1656; found, 392.1647.

Compound **2**: [α]_D_^20^ = +358.95 (*c* 0.53, CHCl_3_). mp: 67–69 °C. ^1^H NMR (400 MHz, DMSO-*d*_*6*_): *δ* 8.63 (d, *J* = 9.6 Hz, 1 H), 8.59 (d, *J* = 4.8 Hz, 1 H), 7.97 (m, 1 H), 7.59 (m, 3 H), 7.44 (t, *J* = 7.6 Hz, 2 H), 7.36 (d, *J* = 7.6 Hz, 2 H), 7.30 (t, *J* = 7.2 Hz, 1 H), 7.14 (t, *J* = 7.6 Hz, 2 H), 6.99 (t, *J* = 7.2 Hz, 1 H), 5.37 (d, *J* = 9.6 Hz, 1 H), 5.22 (d, *J* = 6.6 Hz, 1 H), 4.88 (d, *J* = 6.6 Hz, 1 H), 4.08 (d, *J* = 11.6 Hz, 1 H), 3.92 (d, *J* = 11.6 Hz, 1 H), 3.31 (s, 1 H). ^13^C NMR (100 MHz, CDCl_3_): *δ* 162.1, 148.1, 146.7, 143.8, 141.1, 138.9, 129.2, 127.9, 127.7, 126.9, 126.7, 126.5, 124.9, 123.3, 89.4, 81.9, 67.6, 48.7. FT-IR *ν*_max_/cm^−1^: 3391, 3059, 2873, 1676, 1592, 1570, 1518, 1169, 1088. ESI-HRMS (*m*/*z*): [M + H]^+^ calcd for C_22_H_21_O_3_N_2_ 361.1547; found, 361.1541.

### Preparation of racemic ethyl 3-(4-(benzyloxy)phenyl)-3-((4-fluoro-phenyl)amino)propanoate (*rac*-3)

To a solution of trichlorosilane (0.14 mL, 1.4 mmol) dissolved in dry DCM (1/4, v/v), a drop of DMF and a solution of compound **1** (117 mg, 0.3 mmol) in dry DCM (3 mL) were added at −15 °C. The reaction mixture was stirred at −10 °C for 10 h, and was then quenched with H_2_O (1 mL). The mixture was extracted with DCM (70 mL) and dried over anhydrous Na_2_SO_4_. The solution was filtered and the solvent was removed under vacuum. The residue was subjected to silica gel chromatography to give *rac*-**3**. *rac*-**3**: mp: 65–66 °C. ^1^H NMR (400 MHz, CDCl_3_): *δ* 7.35 (m, 7 H), 6.94 (m, 2 H), 6.81 (m, 2 H), 6.50 (m, 2 H), 5.03 (s, 2 H), 4.70 (t, *J* = 6.8 Hz, 1 H), 4.11 (q, *J* = 7.2 Hz, 2 H), 2.77 (d, *J* = 6.8 Hz, 2 H), 1.19 (t, *J* = 7.2 Hz, 3 H). ^13^C NMR (100 MHz, CDCl_3_): *δ* 171.2, 158.2, 157.3, 154.9, 143.0, 136.9, 134.1, 128.6, 128.0, 127.5, 127.4, 115.7, 115.5, 115.1, 114.9, 114.8, 70.1, 60.8, 55.3, 42.8, 14.2. FT-IR *ν*_max_/cm^−1^: 3344, 3069, 3029, 1723, 1611, 1511, 1381, 1263. ESI-HRMS (*m*/*z*): [M + H]^+^ calcd for C_24_H_25_O_3_NF 394.1813; found, 394.1804.

The racemate was further separated with a preparative OD-H column with a general procedure to afford the *R* and *S* enantiomers. *R*-(**3**), [α]_D_^20^ = +5.5 (*c* 0.65, CHCl_3_) (lit. [α]_D_^20^ = +6.35 (*c* 0.36, CH_2_Cl_2_), absolute configuration unknown)^27^.

### Quantum chemical calculations

All quantum chemical calculations were performed on (*R*)-**3**. Systematic conformational analysis was performed with the MMFF94 force field in the MOE software package^28^. The 63 conformers obtained were optimized with Gaussian 09 using the B3LYP hybrid functional at the 6–31 G(d) basis set level^29^. These conformers were confirmed as minima on the potential energy surface by showing no imaginary frequency. Oscillator strengths and rotational strengths in the dipole velocity representation of the 100 lowest electronic transitions were calculated for each conformer at the 6–31 + G(d,p) level using the Cam-B3LYP functional. ECD spectra were simulated by using a Gaussian function with band width of σ = 0.35 eV.

### General procedure for orthogonal array design

To a solution of compounds **1** (0.22 mmol) and **2** in dry DCM (1.2 mL), trichlorosilane (0.1 mL, 0.99 mmol) was added. The reaction mixture was stirred under various temperatures, times, and amounts of chiral Lewis base catalyst (Table 1). The reaction was quenched with H_2_O, extracted with DCM, and washed with brine. The organic layer was subjected to RPLC-ECD to afford the reaction yield and optical purity values simultaneously.

## Additional Information

**How to cite this article:** Zhang, X. *et al*. A high-performance liquid chromatography-electronic circular dichroism online method for assessing the absolute enantiomeric excess and conversion ratio of asymmetric reactions. *Sci. Rep.* **7**, 43278; doi: 10.1038/srep43278 (2017).

**Publisher's note:** Springer Nature remains neutral with regard to jurisdictional claims in published maps and institutional affiliations.

## Supplementary Material

Supplementary Information

## Figures and Tables

**Table 1:** The factors and factor levels of orthogonal experiment L_9_(3^4^).

Level	Influencing-factor		
	A^a^	B^b^	C^c^
1	5	−10	8
2	10	0	16
3	20	10	24

^a^Cat. (mol %).

^b^Temperature (°C).

^c^Time (h).

**Table 2:** Results of the orthogonal array experiment.

No.	Factors				
	A^a^	B^b^	C^c^	CR (%)^d^	OP (%)^e^
1	1 (5)	1 (−10)	1 (8)	89.96	93.56
2	1 (5)	2 (0)	2 (16)	93.82	92.60
3	1 (5)	3 (10)	3 (24)	96.32	91.41
4	2 (10)	1 (−10)	2 (16)	99.80	91.59
5	2 (10)	2 (0)	3 (24)	100.00	89.23
6	2 (10)	3 (10)	1 (8)	99.37	88.92
7	3 (20)	1 (−10)	3 (24)	100.00	90.76
8	3 (20)	2 (0)	1 (8)	97.11	89.04
9	3 (20)	3 (10)	2 (16)	100.00	86.72

^a^Catalyst (mol %).

^b^Temperature (°C).

^c^Time (h).

^d^CR: conversion ratio.

^e^OP: optical purity.

**Table 3:** Analysis of the L_9_(3^4^) orthogonal array experiment results.

Range analysis	Factors for CR^d^			Factors for OP^e^		
	A^a^	B^b^	C^c^	A^a^	B^b^	C^c^
M1	93.37	96.59	95.48	92.52	91.97	90.51
M2	99.73	96.98	97.88	89.91	90.29	90.31
M3	99.04	98.56	98.77	88.84	89.01	90.47
R	6.36	1.98	3.29	3.68	2.96	0.20
Factor order	A > C > B			A > B > C		
Optimal level	A_2_	B_3_	C_3_	A_1_	B_1_	C_1_
Optimal combination	A_2_B_3_C_3_			A_1_B_1_C_1_		

^a^Catalyst (mol %).

^b^Temperature (°C).

^c^Time (h).

^d^CR: conversion ratio.

^e^OP: optical purity.
