# Supplementary material for: A high-performance liquid chromatography-electronic circular dichroism online method for assessing the absolute enantiomeric excess and conversion ratio of asymmetric reactions
Source: Sci Rep. 2017 Mar 2;7:43278. doi: 10.1038/srep43278 (PMC5333115; doi:10.1038/srep43278)
Supplement: Supplementary Information [file srep43278-s1.doc]

**Supplementary Information**

**A high-performance liquid chromatography-electronic circular dichroism online method for assessing the absolute enantiomeric excess and conversion ratio of asymmetric reactions**

Xiang Zhang,^†^ Mingchao Wang,^†^ Li Li*and Dali Yin*

State Key Laboratory of Bioactive Substances and Functions of Natural Medicines, Beijing Key Laboratory of Active Substances, Discovery and Drugability Evaluation, Institute of Materia Medica, Peking Union Medical College, Chinese Academy of Medical Sciences, Beijing 100050, P.R. China.

E-mail: annaleelin@imm.ac.cn; yindali@imm.ac.cn; Fax: +86 10 63165248; Tel: +86 10 63165248.

^†^ X. Zhang and M.C. Wang contributed equally to this work; ∗ Corresponding author.

The List of Contents

| No. | Content | Page |
| --- | --- | --- |
| 1 | **General information** | S3 |
| 2 | **Figure S1**. HPLC-ECD analysis results of *rac*-**3**, (*R*)-**3**, and (*S*)-**3** using chiral OD-H column. | S4 |
| 3 | **Figure S2**. Standard curves of (*R*)-**3** and (*S*)-**3** at 250 nm. | S5 |
| 4 | **Figure S3**. *G***-**factors of (*R*)-**3** at different concentrations using an OD-H chiral column. | S6 |
| 5 | **Figure S4**. RPLC-ECD analysis results of the representative conditions using a C18 column | S7 |
| 6 | **Figure S5**. The ^1^H-NMR spectrum of ethyl 3-(4- (benzyloxy)phenyl)-3-((4-fluorophenyl)amino) acrylate (**1**) | S8 |
| 7 | **Figure S6**. The ^13^C-NMR spectrum of **1** | S8 |
| 8 | **Figure S7**. The ^1^H-NMR spectrum of (*S*)-N-(4,4- diphenyl-1,3-dioxan-5-yl)picolinamide ((*S*)-**2**) | S9 |
| 9 | **Figure S8**. The ^13^C-NMR spectrum of (*S*)-**2** | S9 |
| 10 | **Figure S9**. The ^1^H-NMR spectrum of (*R*)-3-(4- (benzyloxy)phenyl)-3-((4-fluorophenyl)amino) propanoate ((*R*)-(**3**)) | S10 |
| 11 | **Figure S10**. The ^13^C-NMR spectrum of (*R*)-(**3**) | S10 |
| 12 | **Figure S11**. The ^1^H-NMR spectrum of *rac*-**3** | S11 |
| 13 | **Figure S12**. The IR spectrum of **1** | S11 |
| 14 | **Figure S13**. The IR spectrum of (*S*)-**2** | S12 |
| 15 | **Figure S14**. The IR spectrum of (*R*)-**3** | S12 |
| 16 | **Figure S15**. The ESI-HRMS spectrum of **1** | S13 |
| 17 | **Figure S16**. The ESI-HRMS spectrum of (*S*)-**2** | S13 |
| 18 | **Figure S17**. The ESI-HRMS spectrum of (*R*)-**3** | S14 |

**General information**

^1^H and ^13^C NMR spectra were recorded on Varian Mercury-400, or Varian Mercury-500 NMR spectrometers in CDCl_3_ or DMSO-*d_6_* using TMS as internal standard. The following multiplicity abbreviations are used: (s) singlet, (d) doublet, (t) triplet, (q) quartet and (m) multiplet. IR spectra were measured on Thermo Nicolet 5700 FT-IR-Microscope. ESI-HRMS data were measured on Thermo Exactive Orbitrap mass spectrometer. HPLC-ECD analysis was carried out using a Jasco LC-2000 HPLC system (MD-2010 diode-array detector, Pu-2089 quaternary gradient pump, As-2055 autosampler), connected with a Jasco CD-2095 detector. The detection wavelength was set at 250 nm. Chiral separation was performed on a Daicel OD-H chiral column (5 μm, 4.6 × 250 mm) at 20°C. The mobile phase is n-hexane and isopropanol in a ratio of 90/10 at a flow rate of 1.0 mL/min. The detection wavelength is set as 250 nm. RPLC-ECD analysis was performed using an Ultimate® XB-C18 column (5 μm, 4.6 × 250 mm). Melting points were determined on a Yanaco MP-J3 microscope melting point apparatus. Anhydrous dichloromethane (DCM) was prepared by ITS (PS-MD-7) solvent purification system. Flash column chromatography was performed on Biotage Isolera One.

####
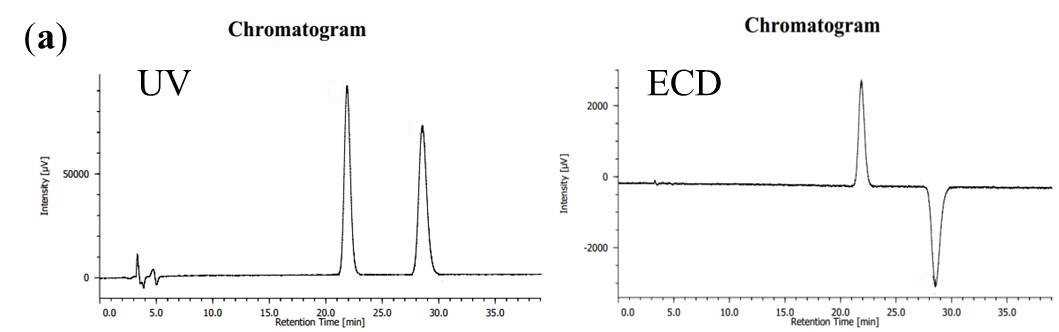


####
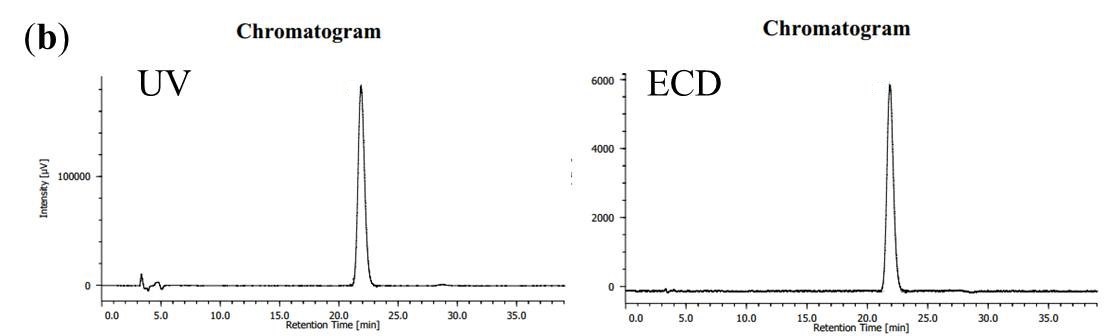


####
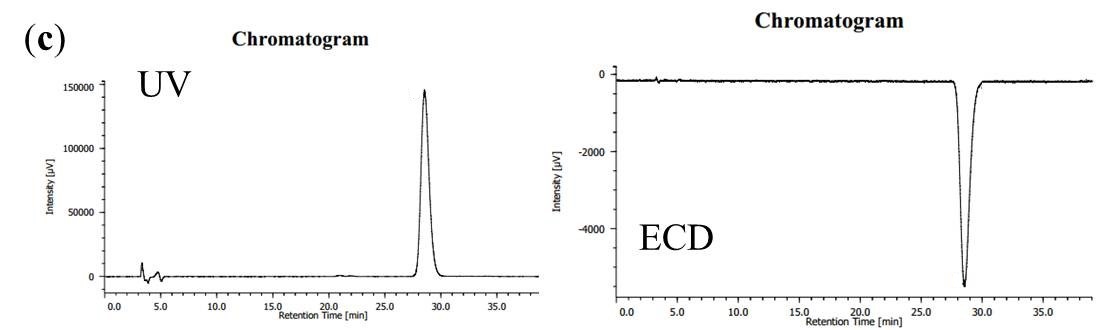


#### Figure S1. HPLC-ECD analysis results of *rac*-3, (*R*)-3, and (*S*)-3 using chiral OD-H column. (a). *rac*-3, (b). (*R*)-3, (c). (*S*)-3.

####
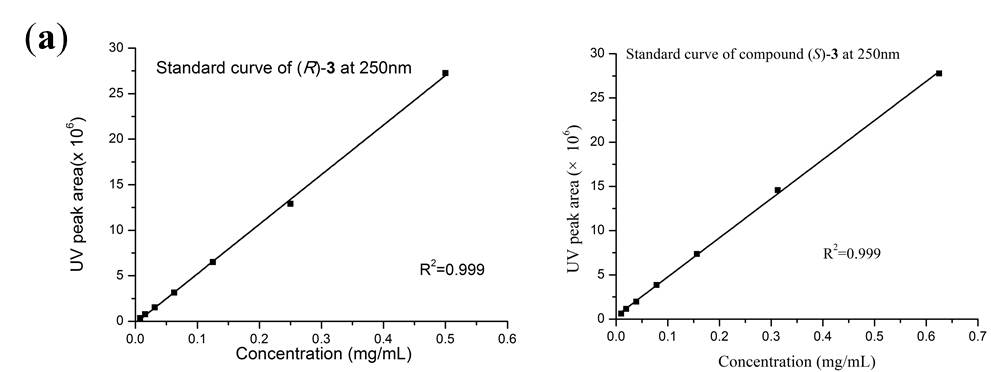


####
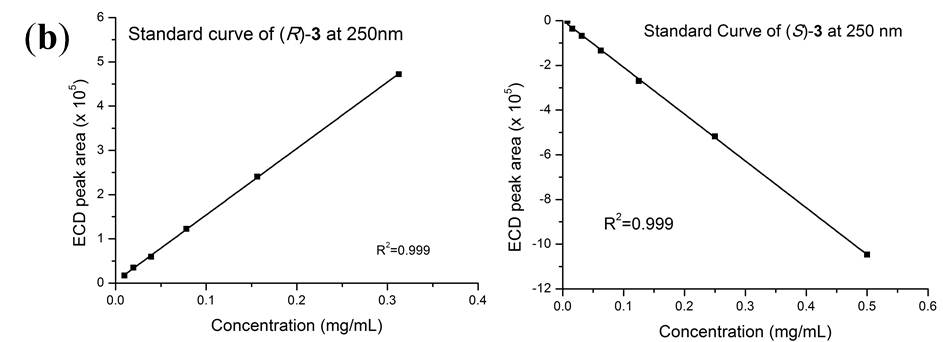


#### Figure S2. Standard curves of (*R*)-3 and (*S*)-3 at 250 nm. (a). UV, (b). ECD.

####
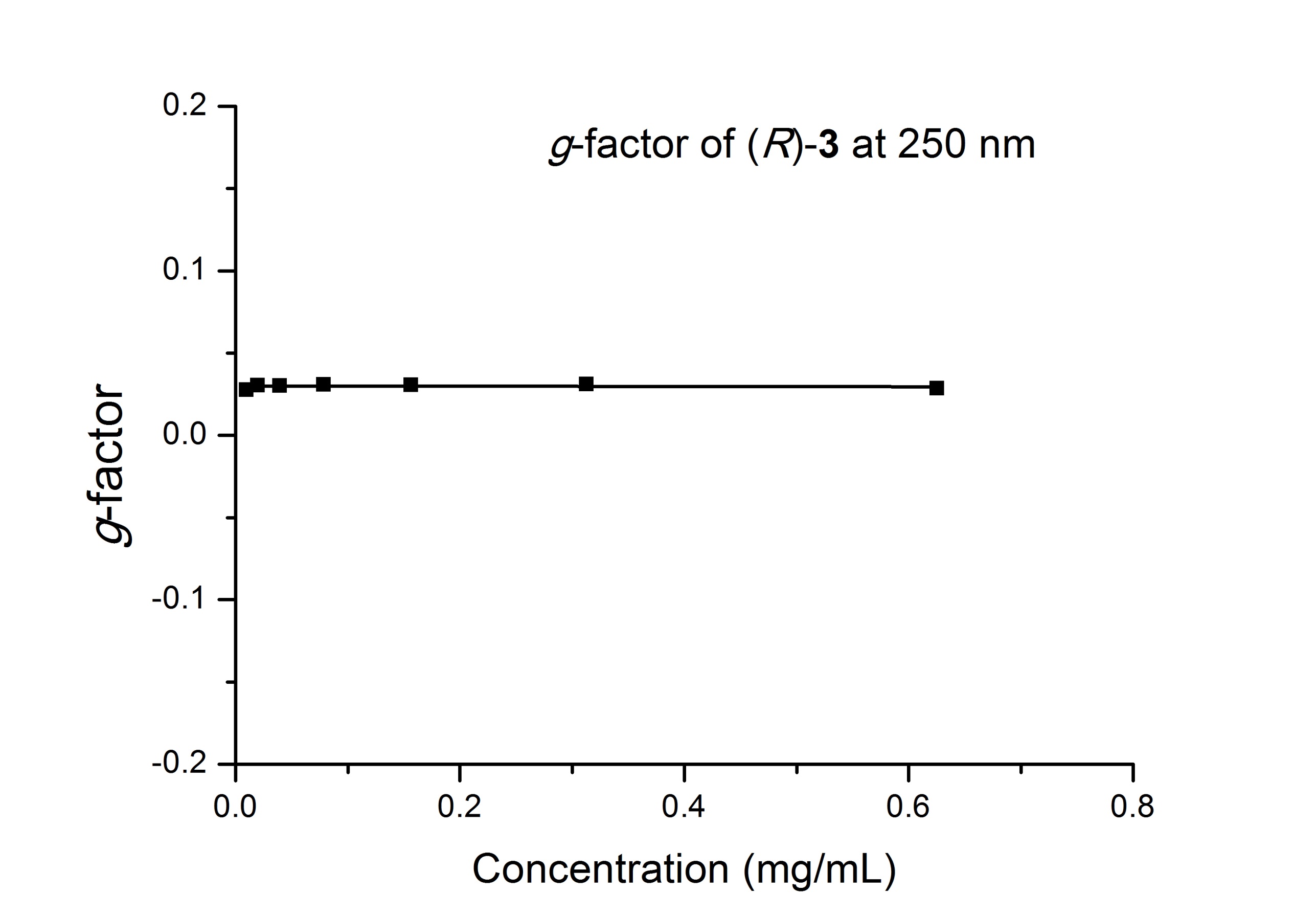


#### Figure S3. *G*-factors of (*R*)-3 at different concentrations using an OD-H chiral column.


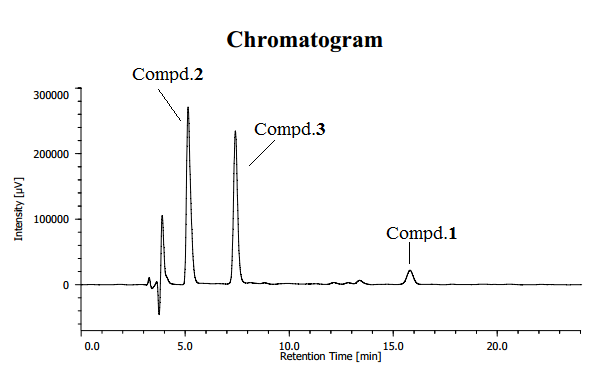

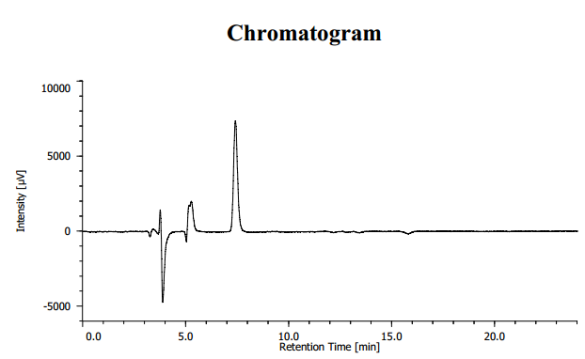


(**a**)

UV

(**b**)

####
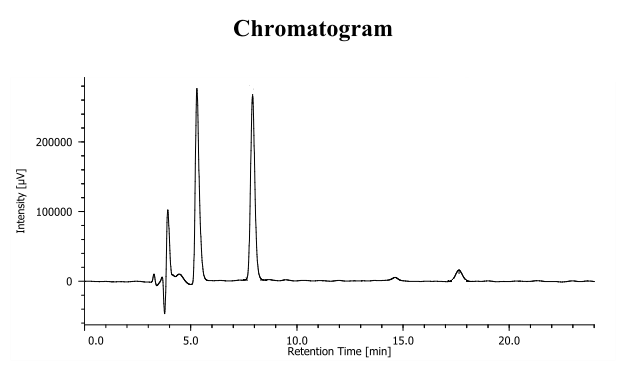

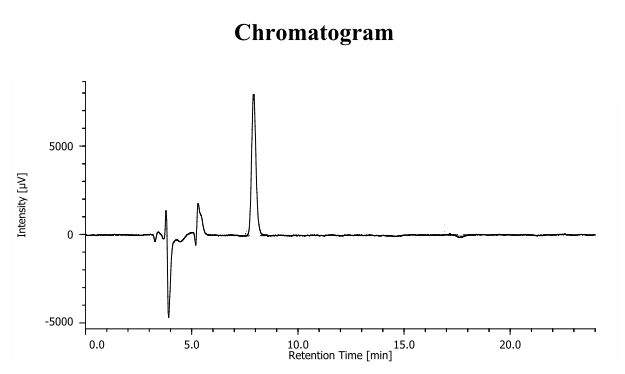


UV

UV

ECD

ECD

ECD

(**c**)

####
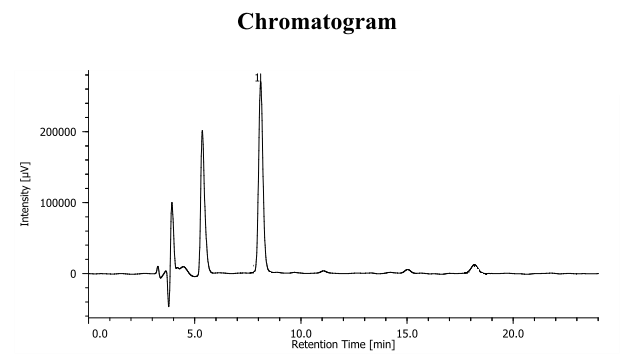

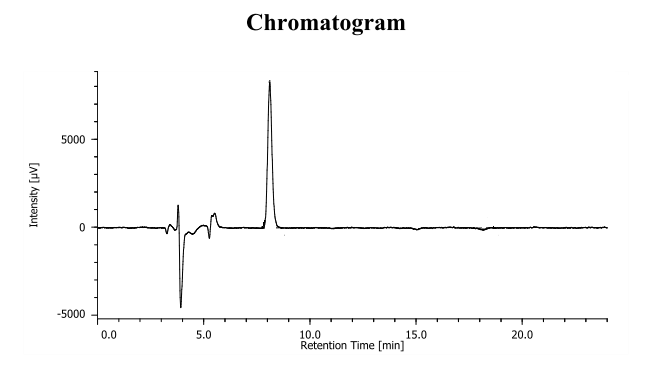


#### Figure S4. RPLC-ECD analysis results of the representative conditions using a C18 column. (a). A_1_B_1_C_1_, (b) A_1_B_2_C_2_, (c). A_1_B_3_C_3_.


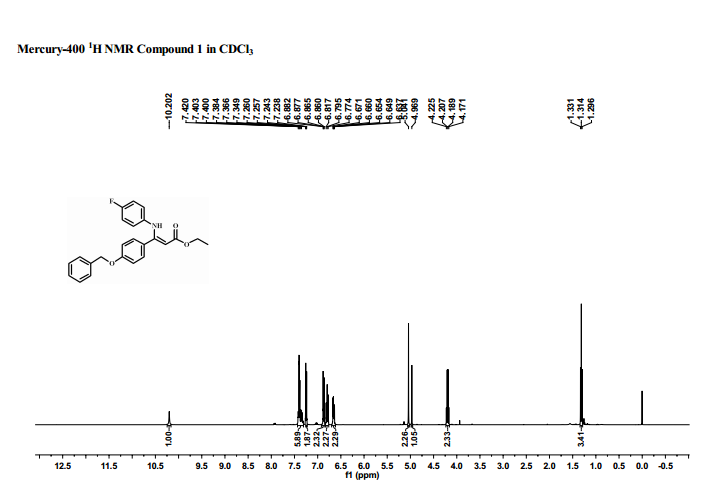


**Figure S5**. The ^1^H-NMR spectrum of ethyl3-(4-(benzyloxy)phenyl)-3- ((4-fluorophenyl)amino) acrylate (**1**)

**
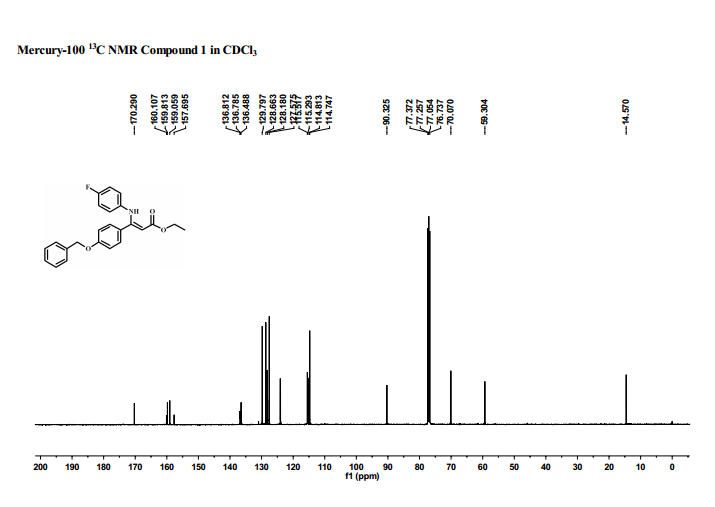
**

**Figure S6**. The ^13^C-NMR spectrum of **1**


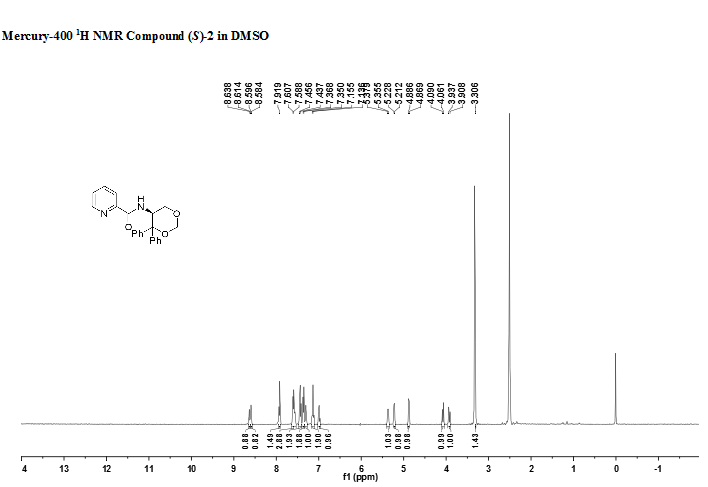


**Figure S7**. The ^1^H-NMR spectrum of (*S*)-N-(4,4-diphenyl-1,3-dioxan- 5-yl)picolinamide ((*S*)-**2**)

**
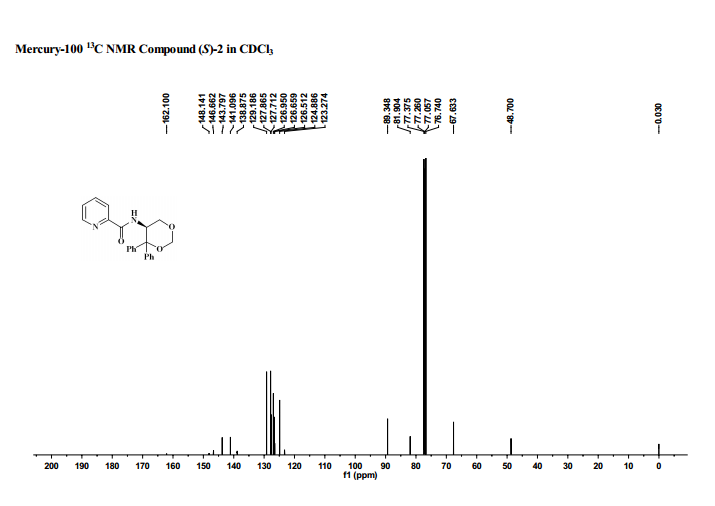
**

**Figure S8**. The ^13^C-NMR spectrum of (*S*)-**2**

**
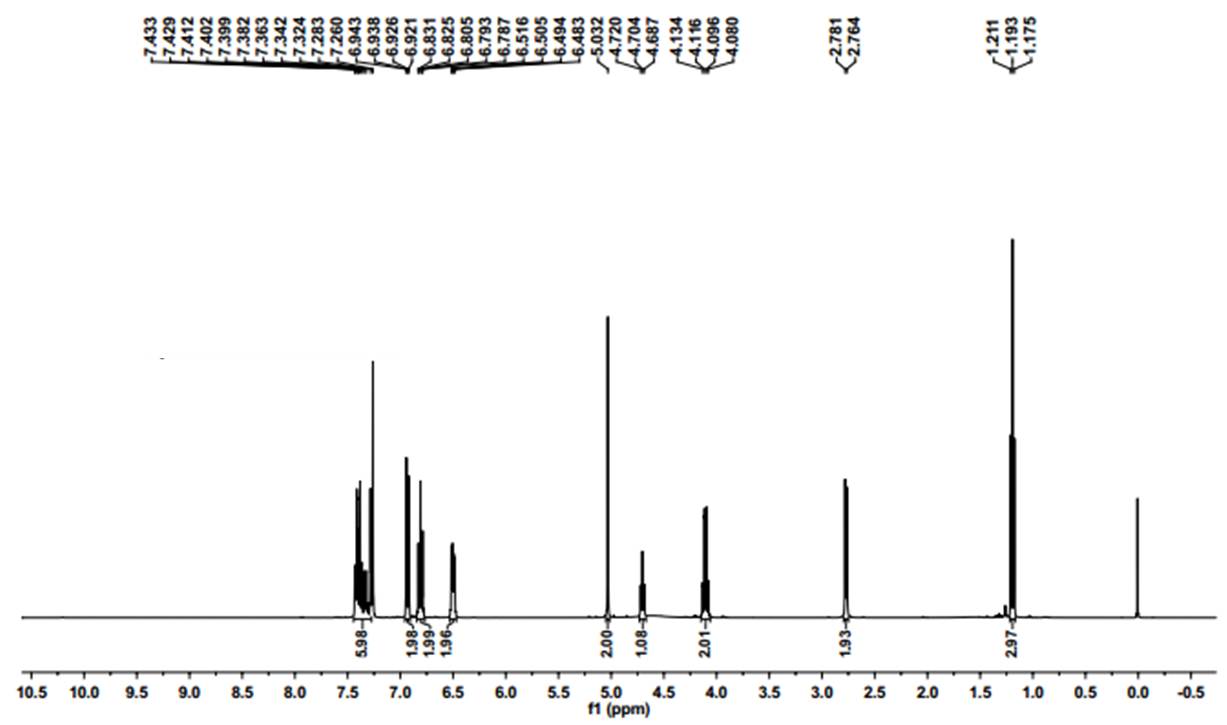
**

**Figure S9**. The ^1^H-NMR spectrum of (*R*)-3-(4-(benzyloxy)phenyl)-3- ((4-fluorophenyl) amino)propanoate ((*R*)-**3**)

**
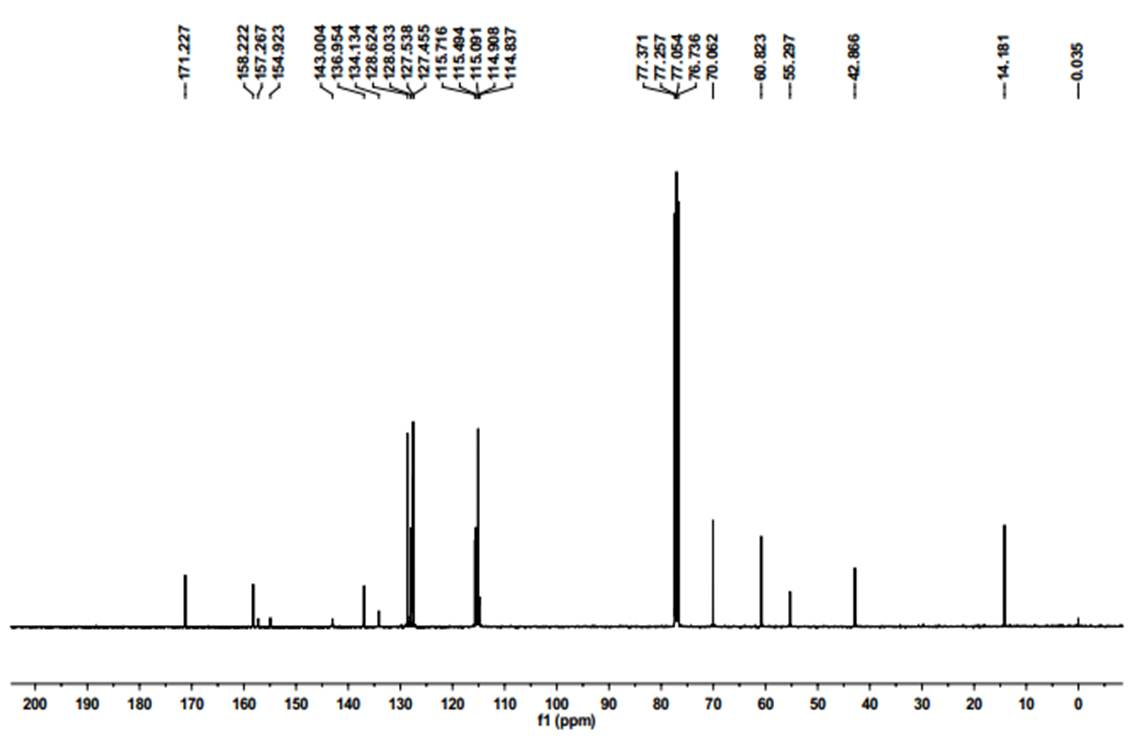
**

**Figure S10**. The ^13^C-NMR spectrum of (*R*)-**3**


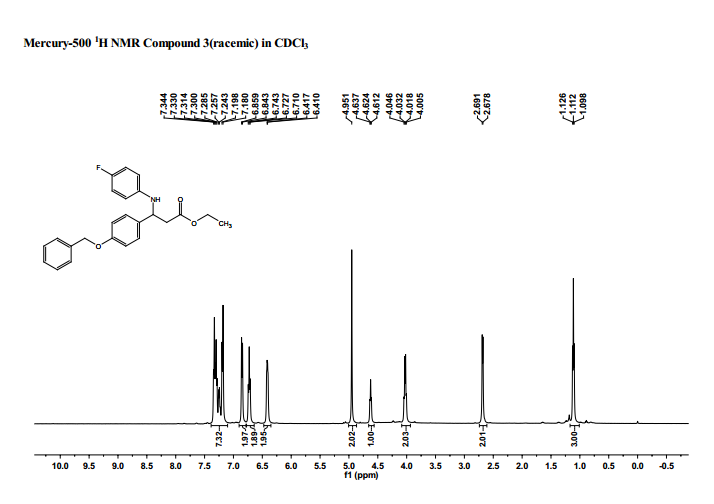


**Figure S11**. The ^1^H-NMR spectrum of *rac*-**3**


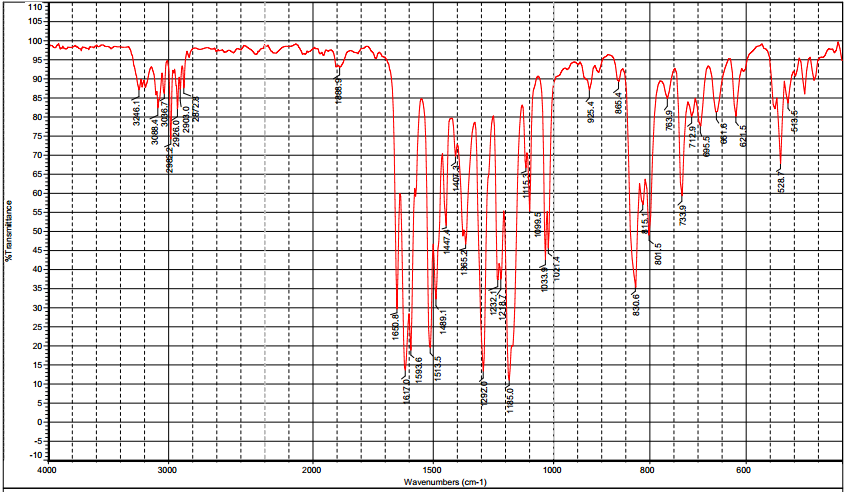


**Figure S12**. The IR spectrum of **1**


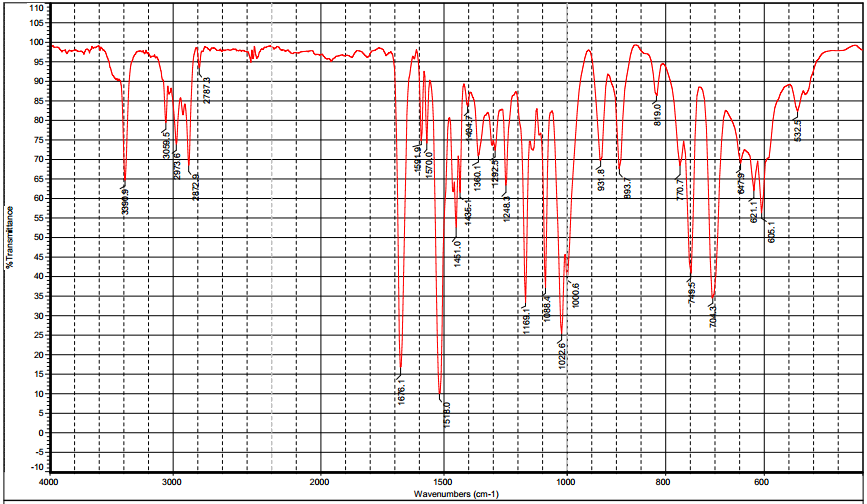


**Figure S13**. The IR spectrum of (*S*)-**2**


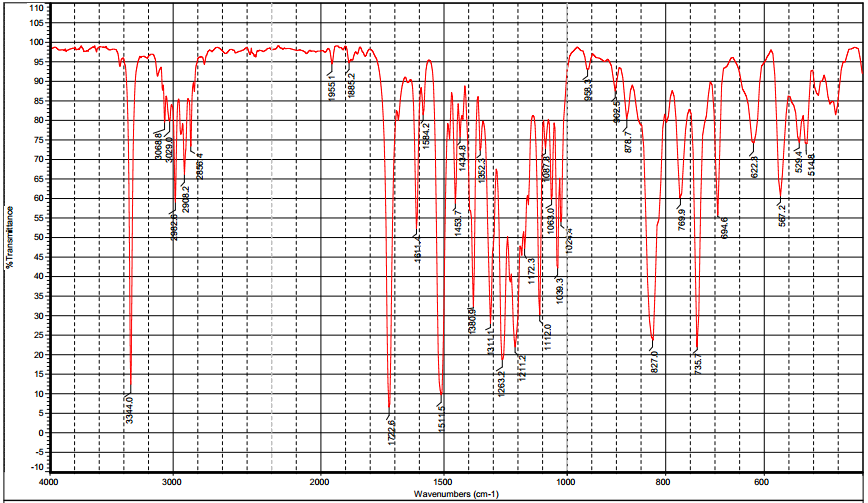


**Figure S14**. The IR spectrum of (*R*)-**3**


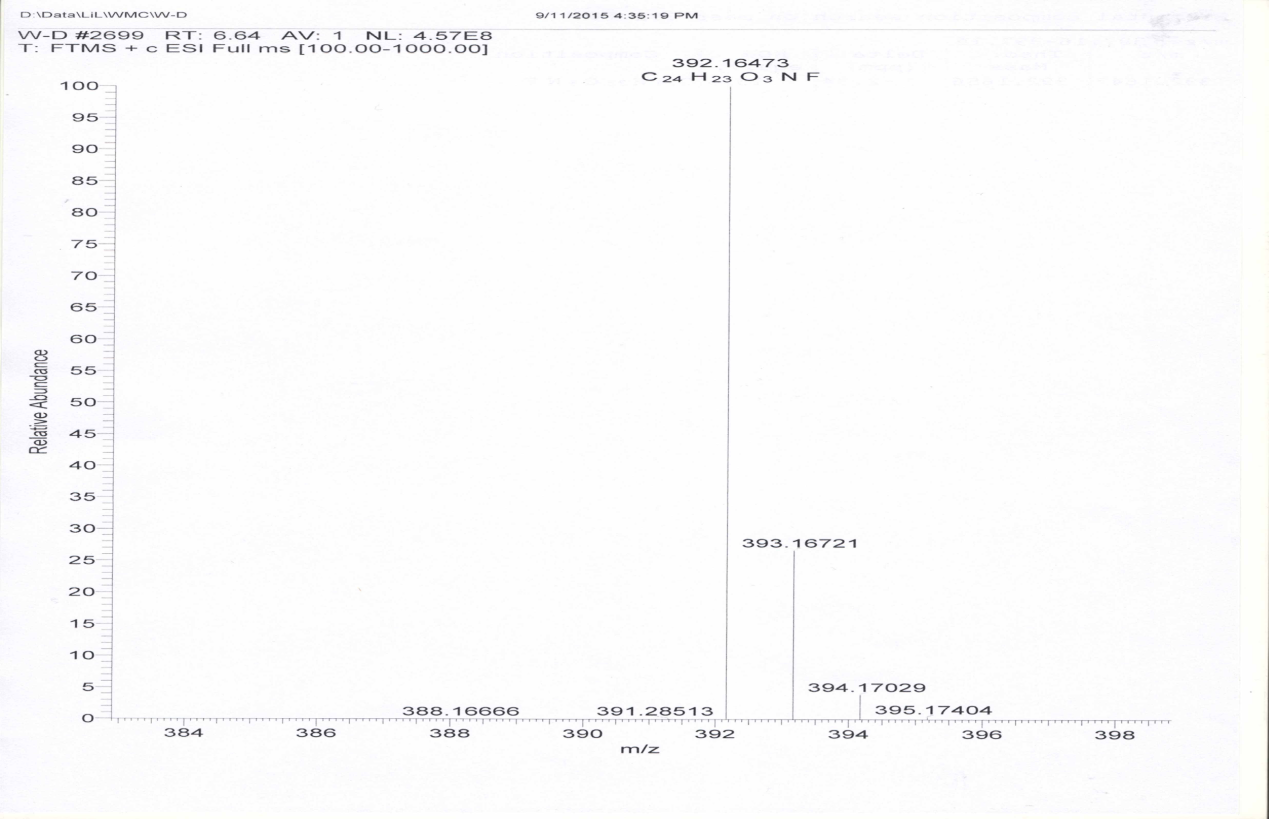


**Figure S15**. The ESI-HRMS spectrum of **1**


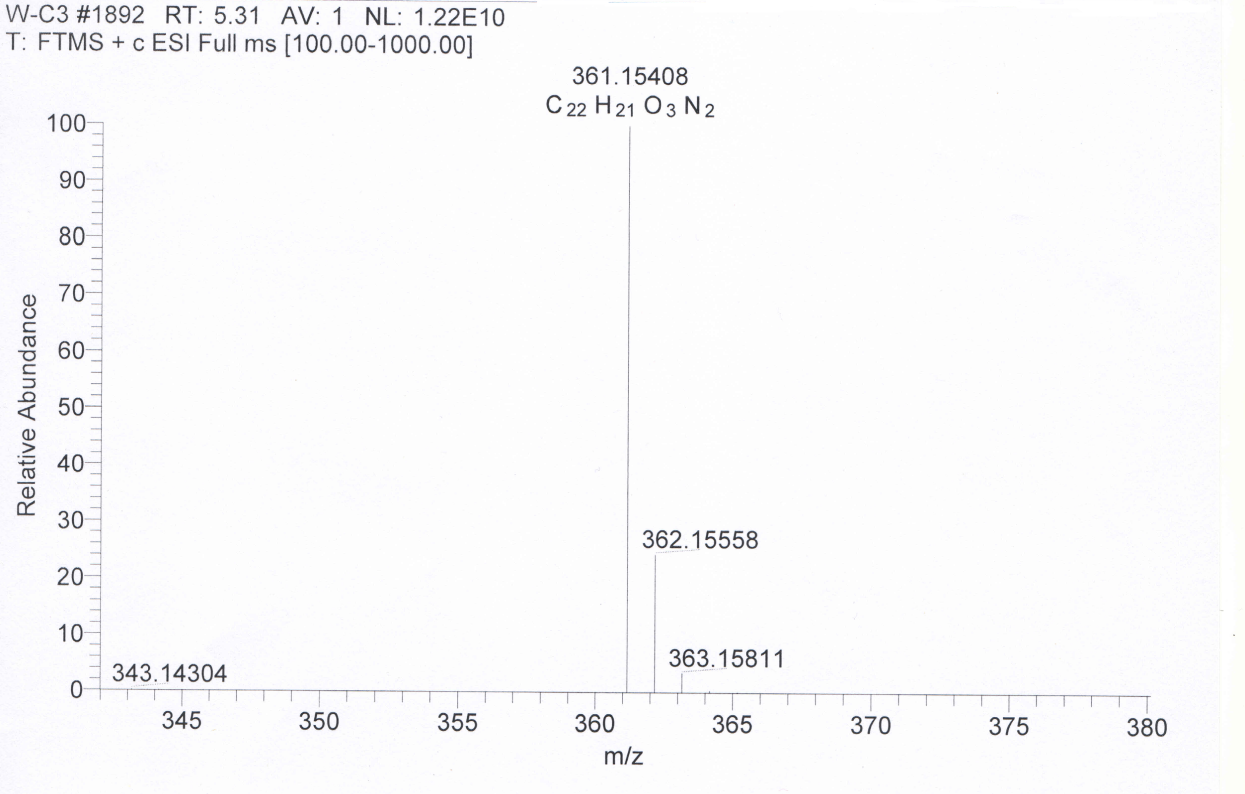


**Figure S16**. The ESI-HRMS spectrum of (*S*)-**2**


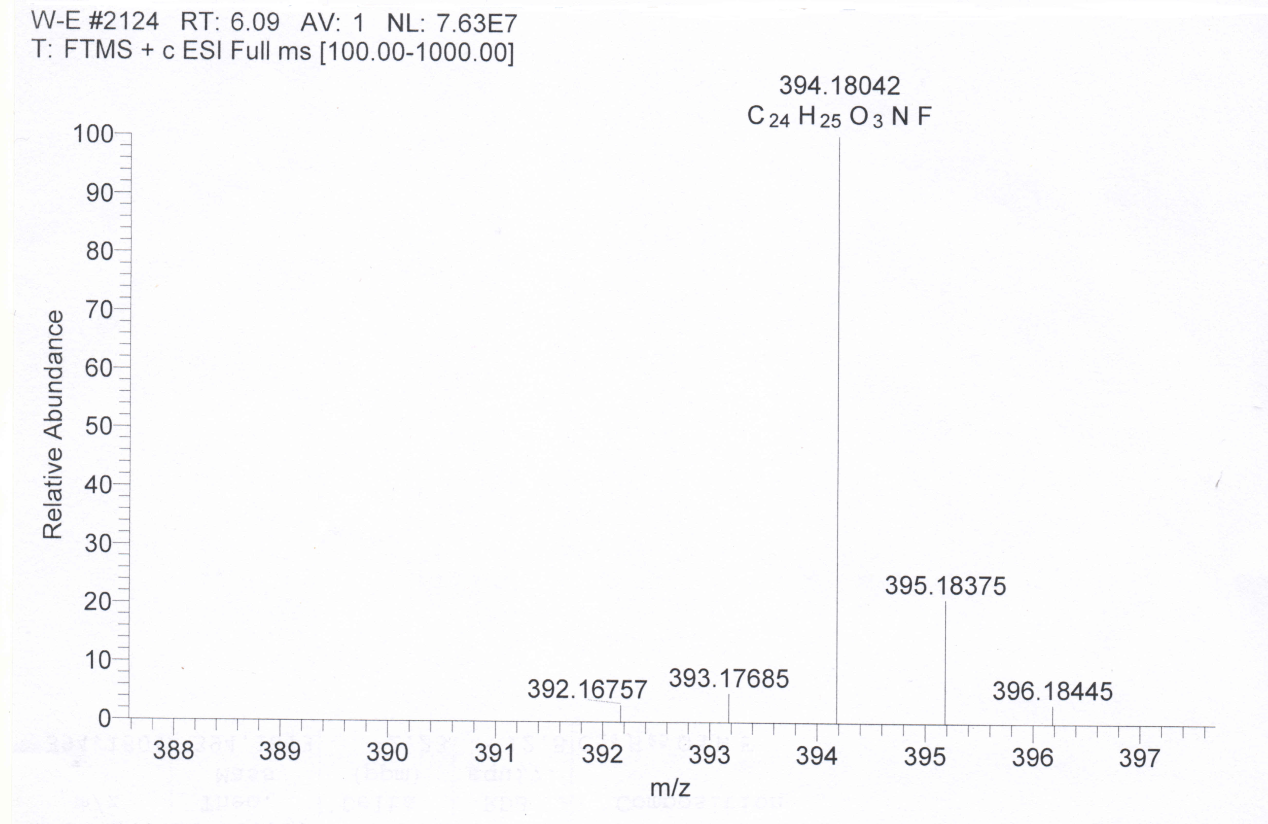


**Figure S17**. The ESI-HRMS spectrum of (*R*)-**3**
